# Cap-assisted photodynamic therapy for esophageal cancer: precise irradiation field control for tumor-confined treatment

**DOI:** 10.1055/a-2967-7860

**Published:** 2026-10-12

**Authors:** Noriaki Sugawara, Taro Iwatsubo, Kazuki Takayama, Shun Sasaki, Akitoshi Hakoda, Kazuhiro Ota, Hiroki Nishikawa

**Affiliations:** 1 2nd Department of Internal Medicine 13010 Osaka Medical and Pharmaceutical UniversityTakatsukiOsaka PrefectureJapan; 2 Endoscopy Center 38588 Osaka Medical and Pharmaceutical University HospitalTakatsukiOsaka PrefectureJapan

Photodynamic therapy (PDT) is a safe and effective salvage treatment for local recurrence after chemoradiotherapy or radiotherapy for esophageal cancer. 1​ However, adverse events such as esophageal stricture and perforation may occur. 2​ The stricture is of particular concern as the risk increases with the circumferential extent of the lesion. To minimize this risk, avoiding unnecessary irradiation of the surrounding normal mucosa is important.

In PDT, laser light is delivered endoscopically to the lesion after intravenous administration of a photosensitizer. A cap is attached to the endoscope tip to control the direction of irradiation; however, the cap does not fit against the esophageal mucosa, resulting in light leakage and an inconsistent irradiation distance. To overcome this limitation, we developed cap-assisted PDT (cap-PDT), wherein irradiation is delivered through a transparent cap mounted on the endoscope tip.

The cap used for EMR-C 3​ has an obliquely shaped tip that allows device positioning en face with the esophageal mucosa. For cap-PDT, the cap is covered with black tape and oriented such that the oblique opening faces the 12 o’clock direction of the endoscopic view. This configuration enables the en face visualization of the lesion with an up-angulation. The cap prevents laser light leakage, thereby shielding the surrounding normal tissue, minimizing unnecessary mucosal injury, and confining adequate irradiation to the tumor ( Figs. 1 2 ). In addition, contact between the cap opening and the mucosa stabilizes the probe position and maintains an optimal irradiation distance.

**Fig. 1:**
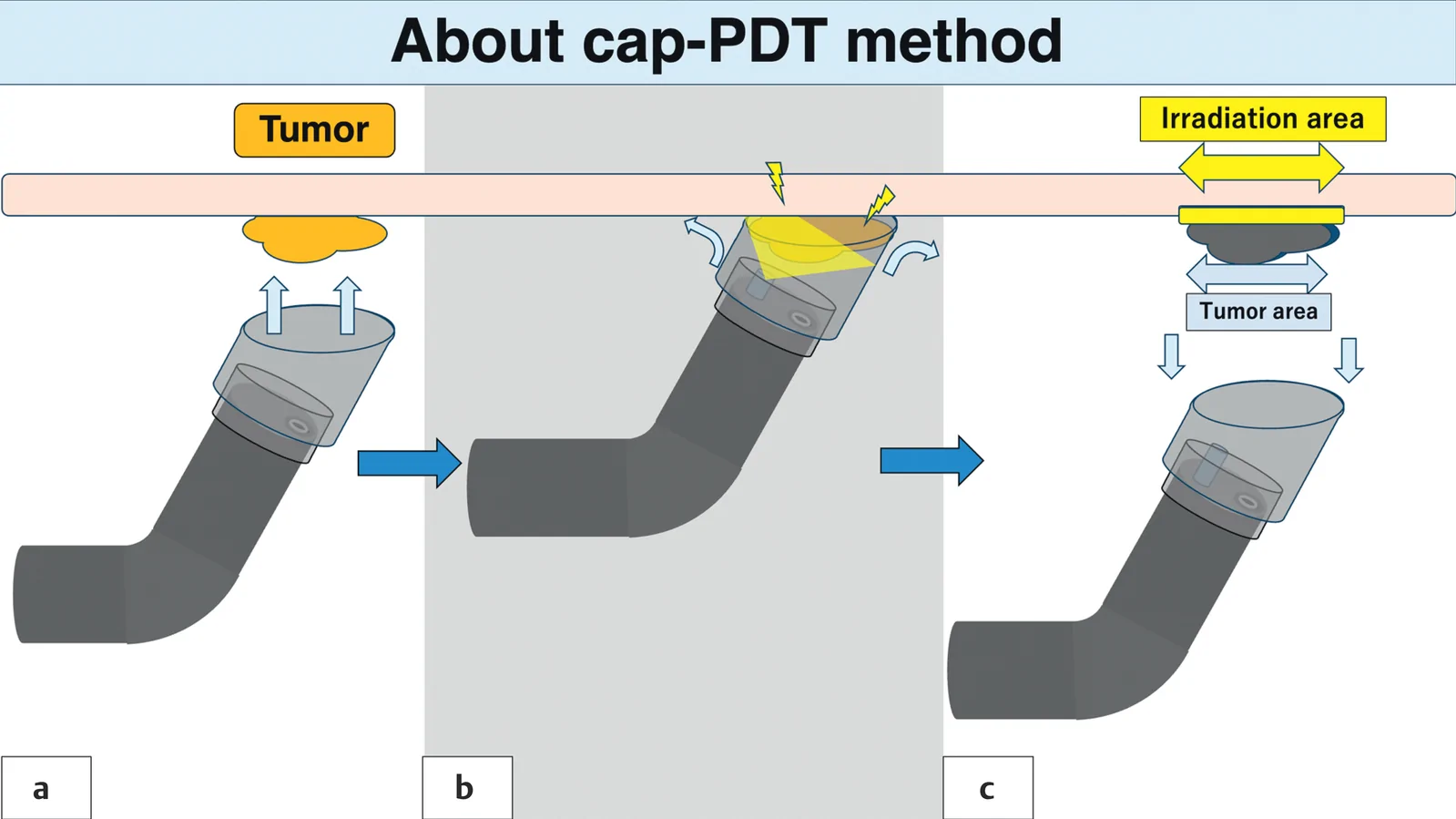
Schematic illustration of the cap-PDT technique. ( **a** ) An EMR-C cap covered with black tape is mounted on the endoscope tip, and the tumor is approached en face. ( **b** ) The cap is placed over the tumor, and laser irradiation is performed. ( **c** ) Irradiation is confined to the tumor area, sparing the surrounding normal mucosa.

**Fig. 2:**
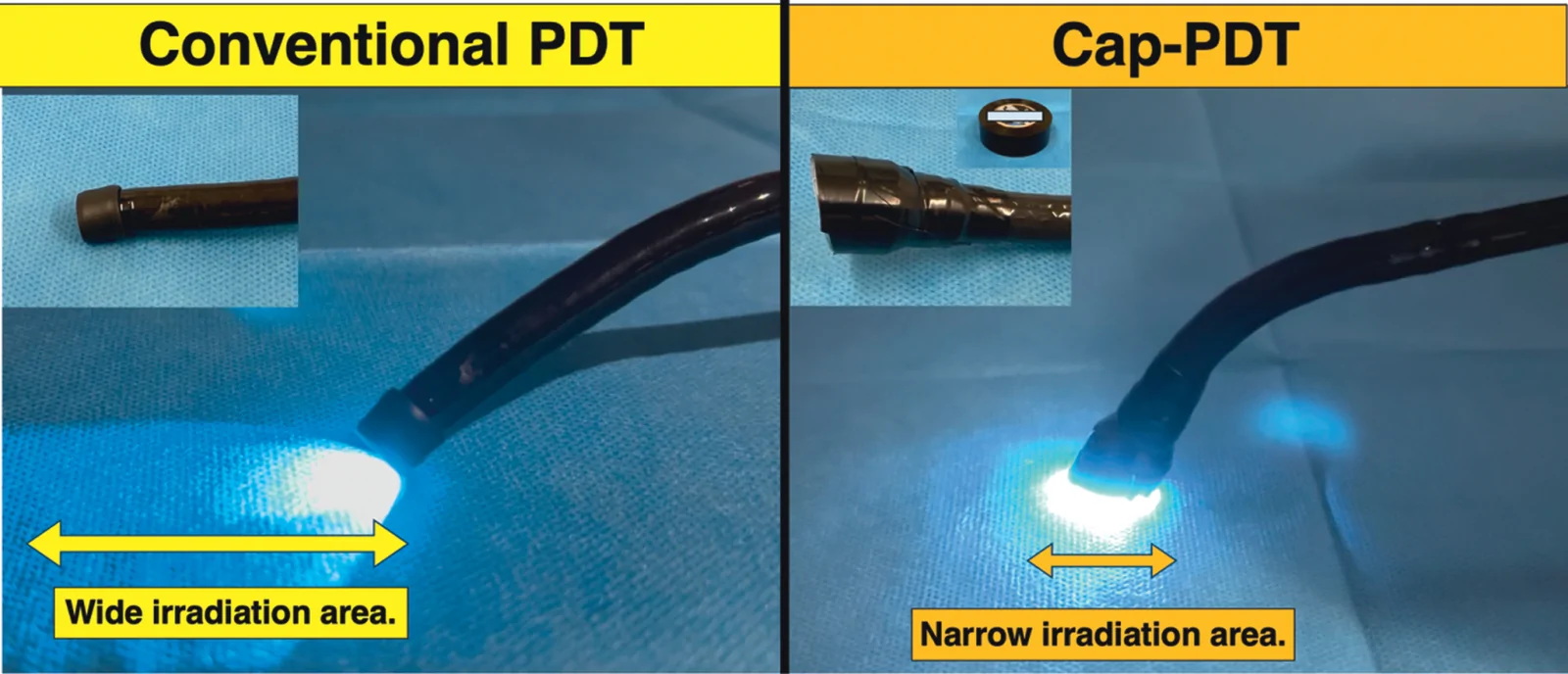
Comparison of the irradiation field between conventional PDT with a black hood and cap-PDT in a simulated setting. Compared with conventional PDT, the irradiation field of cap-PDT was controlled within a narrower area.

Herein, we present a case treated with cap-PDT ( Video 1 ). A 72-year-old man underwent definitive chemoradiotherapy for middle thoracic esophageal cancer. Although the tumor initially resolved, a superficial esophageal carcinoma recurred at the same site 6 years later. The invasion depth was estimated to be T1a-EP/LPM; however, endoscopic resection was considered difficult because of post-chemoradiotherapy fibrosis and adjacent scarring from previous endoscopic treatment ( Fig. 3 ). PDT was therefore performed as salvage therapy. After intravenous talaporfin sodium, the lesion was irradiated at 664 nm and 100 J/cm ^2^ with complete tumor coverage (total energy: 200 J; two sessions). The procedure was completed without adverse events. The lesion healed with scarring at 8 weeks, and no recurrence was observed.

**Video 1** Cap-assisted photodynamic therapy for esophageal cancer, demonstrating cap preparation, en face targeting, and tumor-confined laser irradiation sparing the adjacent normal mucosa. Video URI:


**Fig. 3:**
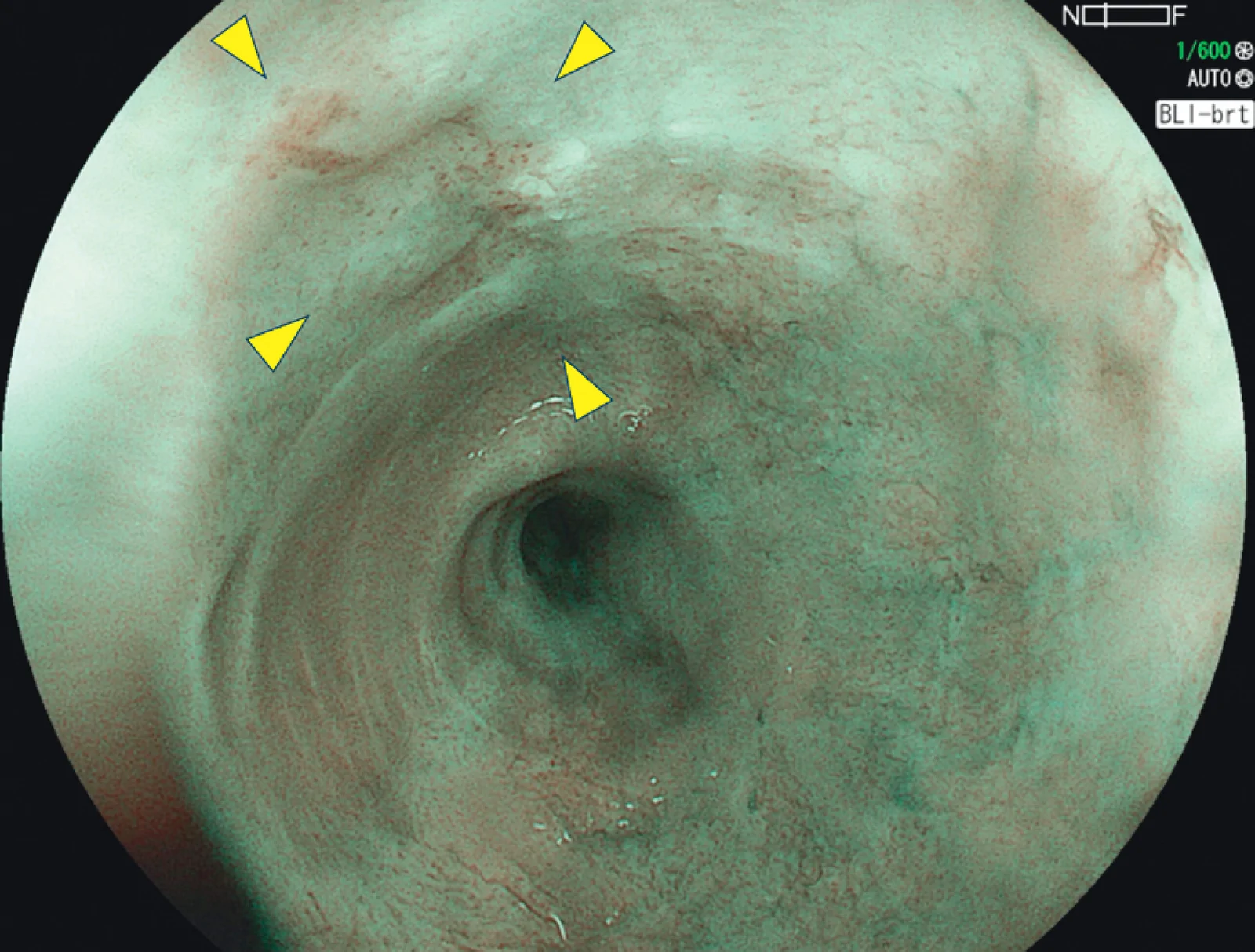
An endoscopic image showing a 0-IIc lesion involving approximately one-quarter of the circumference in the middle thoracic esophagus (yellow arrowheads). The invasion depth was estimated to be T1a-EP/LPM; however, endoscopic resection was considered difficult due to multiple adjacent scars from previous treatment.

Cap-PDT requires simple preparation and enables the controlled delivery of laser light to the target area ( Fig. 4 ). This technique may help reduce the risk of post-procedural esophageal stricture by preventing irradiation of the surrounding normal tissue.

**Fig. 4:**
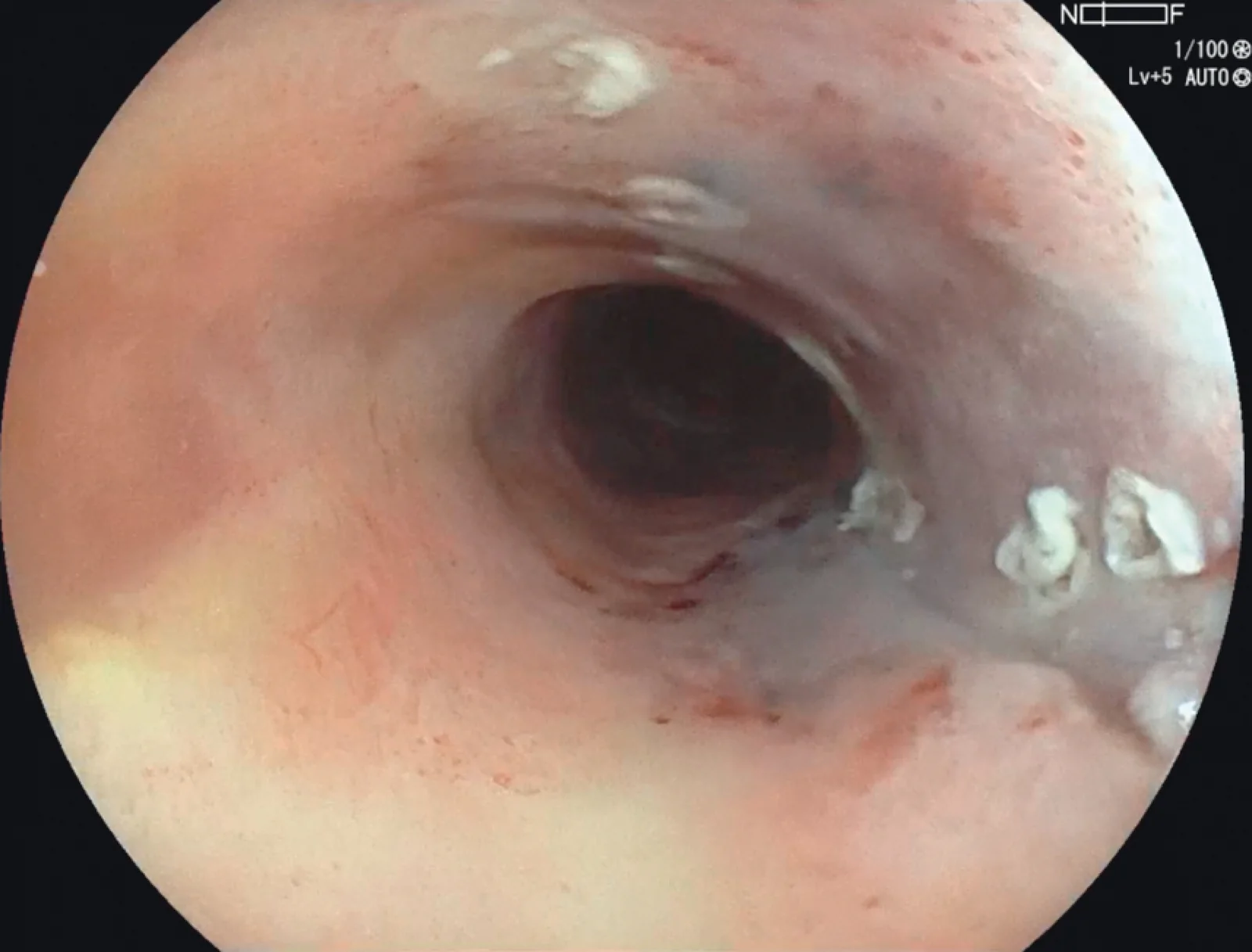
An endoscopic image on the day after PDT. The irradiated area showed a bluish-purple discoloration, indicating sufficient irradiation without excess.

Endoscopy_UCTN_Code_TTT_1AO_2AN

## Declaration of GenAI Use

The authors used a large language model (Claude, Anthropic) to assist with English translation and language editing of the manuscript. All contents were reviewed and verified by the authors, who take full responsibility for the final manuscript.
